# A Cross-Sectional Study on Self-Medication Prevalence and Usage Patterns: An Alarming Concept Among the Saudi Population

**DOI:** 10.7759/cureus.40436

**Published:** 2023-06-14

**Authors:** Abdul Salam Thekkiniyakath Ali, Abdulrahman Abdulelah A Alsheraihi, Saed Saeed Ibrahim Alghamdi, Rahaf Sulaiman Alsuwaylihi, Suha Sulaiman Alenazi, Lamia Saad Bin Hussain

**Affiliations:** 1 Preventive Dental Sciences Department, College of Dentistry, King Saud bin Abdulaziz University for Health Sciences, Riyadh, SAU; 2 Dentistry, College of Dentistry, King Saud bin Abdulaziz University for Health Sciences, Riyadh, SAU

**Keywords:** saudi population, irrational drug use, over the counter drugs, generic drugs, alternative medicines

## Abstract

Background: Self-medication (SM) has many potential drawbacks, including toxicity, drug resistance, severe adverse effects, drug interactions, drug abuse, and drug dependence, but it is still widely used for a variety of reasons, including time and money savings, symptom prevention or treatment of minor illnesses, a lack of access to doctors, etc. This study aimed to describe and analyse the usage of antibiotics without a prescription, self-medication practises, and patterns of using them among Saudis.

Materials and methods: In 13 provinces of Saudi Arabia, a community-based cross-sectional study was done. After gaining consent, data were gathered from 420 adults during face-to-face interviews using a questionnaire. SPSS was used to analyse the data once it had been entered into Microsoft Excel (Microsoft® Corp., Redmond, WA).

Results: Regarding self-medication, among 208 participants, there was a male predominance and among upper lower class with respect to socioeconomic status. The self-medication rate was found to be higher among graduates and professionals with respect to education and occupation. Self-medication was discovered to be more prevalent among metropolitan residents. The majority of those who started using self-medication got their knowledge from various advertisements. The most frequent symptom of self-medication was fever, followed by a common cold. The most commonly used medication was paracetamol, followed by cough syrups. Weight loss advertisements influence participants the most for self-medication, followed by hair loss and diabetes. TV advertisements have the highest influence on people practising self-medication.

Conclusion: The study calls for greater knowledge of the impacts of antibiotic self-medication, which can be accomplished through efficient measures including behaviour change communication and encouraging more research into its causes and effects.

## Introduction

The World Health Organization (WHO) [1] and the International Pharmaceutical Federation (FIP) [2] define self-medication (SM) as a practise by which an individual selects and uses medicines to treat symptoms or minor health problems, recognized as such by themselves. Self-medication is a form of self-care that the WHO recognizes as being beneficial to an individual's health when carried out properly [1-4]. SM is a phenomenon with a far more nuanced description than is currently being suggested. It is a sophisticated behaviour with an evolutionary and adaptive background [5]. Self-medication has historically been defined as "using drugs, plants, or home remedies on one's own initiative or on the advice of another person without seeking medical advice" [6]. Purchase and use of over-the-counter (OTC), prescription-only (POM), and expired medication are all examples of self-medication [7]. Self-medication has a number of negative effects, including resource waste, greater disease resistance, and drug resistance.

Self-medication has also been linked to incorrect dosage, poor administration techniques, prolonged use, improper storage, drug interactions, polypharmacy, and the possibility of dependence and addiction, making it a serious public health issue globally [8]. The risk of morbidity and death may increase as a result of resistance to readily accessible and reasonably priced antimicrobial medicines, thus limiting the already constrained therapeutic options in the management of prevalent infectious illnesses in developing countries [9]. Young people who believe that Google knows everything, daily wage labourers, monthly wage workers, and busy businessmen make up the majority of internet users nowadays. Lack of time, convenience, and awareness parameters, as well as distorted thinking, all contribute to today's never-ending cycle of self-medication and its detrimental effects [10]. The aim of the current study was to analyse the frequency of self-medication among working-age individuals as well as the adverse drug reactions that occur and how severe they are. It is anticipated that the findings will support legislative and policy changes aimed at encouraging appropriate self-medication.

## Materials and methods

Study design

This research was a continuation of already published research on dental myths [11] among the adult population in Saudi Arabia with IRB Number SP21R/363/06 conducted between the months of August and October 2021 and had a cross-sectional design. By using a simple random selection process, the Saudi provinces were chosen, and 26 locations, two from each of the 13 provinces, consisting of 13 urban and 13 rural areas, were selected. Every third house was included in the study after a house listing was conducted in the chosen colonies with the assistance of native dental researchers in the concerned areas.

Data collection

Using a pretested questionnaire, data were gathered through in-person interviews with trained staff. The two elder members of each chosen house who were present at the time of the visit served as the study's subjects. Following three consecutive attempts to contact any unreachable members of the chosen house, the next house was included in the research. In addition to those who declined to take part in the study, registered medical professionals, pharmacists, nurses, and paramedics were also excluded from the analysis. These excluded individuals also included those with severe physical illnesses or who were non-ambulatory and unable to comprehend or respond to questionnaires.

The sample size was determined by the following criteria: confidence interval (1-α) of 95%, absolute precision (d) of 15%, and proportion of antibiotic self-medication (p) of 42% from the previous study [12]. The sample size calculated was 420. A questionnaire was created after a thorough literature search to gather demographic data on age, gender, education, and income. Both open-ended and closed-ended questions were used to get a better idea of the respondents' knowledge, attitudes, and behaviour regarding self-medication, including their pattern of using antibiotics, their reasons for doing so, whether they were influenced by the media in doing so, and their source of medications. The face value of the questionnaire was 1, and the Cronbach's alpha value was 0.8. To weed out unclear questions, 50 questionnaires were pilot-tested after being translated into Arabic. The participants in the pilot study were not included in the final sample.

Statistical analysis

IBM's Statistical Package for Social Sciences (SPSS, IBM Corp., Armonk, NY) version 21 was used to analyse the data. Percentages and proportions were used to represent variables. For drawing statistical inferences, the Chi-square test was utilized, and P < 0.05 was regarded as significant.

## Results

Among the 420 study participants, 215 (51.9%) were male. On educational background, 260 (61.90%) were graduates. Among the participants with regard to socioeconomic status, 209 (49.76%) belong to the upper middle socioeconomic class. Most of them were professionals. Regarding self-medication, among the 208 participants, there was a male predominance and among upper lower class with respect to socioeconomic status. The self-medication rate was found to be higher among graduates and professionals with respect to education and occupation. Self-medication was discovered to be more prevalent among non-Saudi residents (Table 1).

**Table 1:** Distribution of research participants based on socio-demographic characteristics (N=420, n=208)

Variables		Frequency		Self medication rate		P-value
		N	%	n	%	0.201
Gender	Males	215	51.19	113	54.32	
	Females	205	48.81	95	45.67	
Socioeconomic status	Affluent	152	36.19	63	30.28	0.189
	Upper middle	209	49.76	109	52.40	
	Lower middle	28	6.66	17	8.17	
	Deprived class	31	7.38	19	9.13	
Occupation	Seeking work/not working	32	7.61	7	21.87	0.001
	Working- full time	147	35.00	95	64.62	
	Working-part time	132	31.42	60	45.45	
	Retired	109	25.95	46	42.20	
Education	Upto high school (pre and middle school)	89	21.19	48	53.93	0.003
	High school	52	12.38	9	17.31	
	University	260	61.90	146	56.15	
	Illiterate	15	3.57	5	33.33	
Nationality	Saudi	210	50	55	26.19	0.000
	Non-saudi	210	50	153	72.85	

The majority (88.81%) agreed that antibiotics could be purchased over the counter without a prescription from a doctor and recognized antibiotic resistance as a serious public health issue. Interestingly, over 87% believe that antibiotics can also be used to treat viral infections. A larger percentage of participants (68.33% and 47.3%, respectively) believed that newer, more expensive medications were more effective (Table 2).

**Table 2:** Opinions on the use of antibiotic

Comments	Yes		No		Don’t know	
	n	%	n	%	n	%
Can I get antibiotics at the pharmacy without a prescription from a doctor?	373	88.81	33	7.85	14	6.73
Is antibiotic resistance a serious issue for the public health of our country?	303	72.14	93	22.14	24	5.71
Can antibiotics be used to treat viral infections?	365	86.90	40	9.52	15	3.57
Does a greater price indicate a better product?	199	47.3	128	30.47	93	22.14
Are newer antibiotics more effective?	287	68.33	96	22.85	37	8.81

The majority (49.51%) of those who started using self-medication got their knowledge from various adverts. The most frequent symptom for self-medication was fever (49.51%), followed by a common cold (27.4%). The most commonly used medication was antipyretics, followed by cough syrups (Table 3).

**Table 3:** Distribution of research participants based on the use of self-medication (n=208)

Self-medication practices	Frequency	
	n	%
(1) Informational sources		
Previous experience	19	9.13
Old prescriptions	13	6.25
Suggested by pharmacist	14	6.73
Suggested by family members	3	1.44
Suggested by friends	9	4.32
Advertisements	103	49.51
From search engines (Google, etc.)	47	22.59
(2) Self-medication used for		
Common cold	57	27.40
Fever	103	49.51
Sore throat	19	9.13
Running nose	15	7.21
Urinary tract infection	5	2.40
Pains and cramps	3	1.44
Diarrhoea	2	0.96
Vomiting	3	1.44
Small wounds	1	0.48
(3) Drugs commonly used		
Antipyretic	97	46.63
Antiallergic	19	9.13
Cough syrups	38	18.26
Antibiotics	15	7.21
Antacid (any type of digestive syrups)	13	6.25
Contraceptives	17	8.17
Diarrhoea and constipation drugs	9	4.32

**Figure 1:**
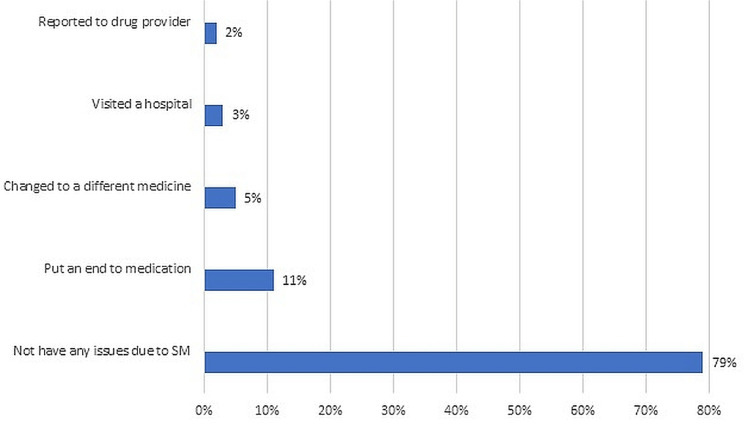
Percentage of people’s response to a drug-related issue brought on by self medication

The majority of the participants practising self-medication reported that they have not faced any health issues due to it; 11% put an end to the medication after they encountered an issue related to self-medication (Figure 1).

**Figure 2:**
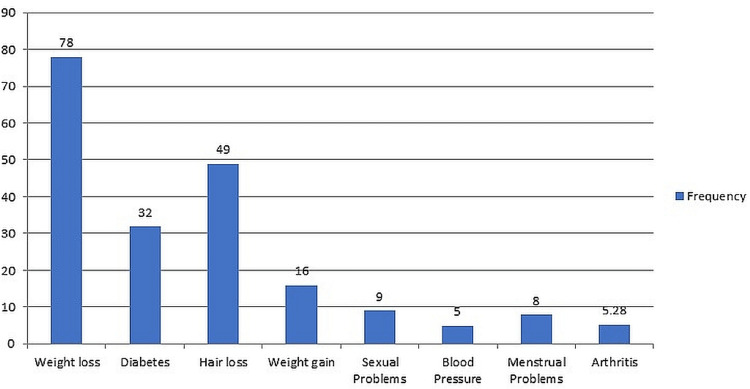
Distribution of participants practising self-medication for different ailments under the influence of advertisement

Weight loss advertisements influence participants the most towards self-medication, followed by hair loss and diabetes (Figure 2).

TV advertisements (26.44%) have the highest influence among people practising self-medication. Newspapers (19.71%) and YouTube (18.75%) also had an impact on the participants' decision to pursue self-medication (Table 4).

**Table 4:** Distribution of participants based on various media sources for self-medication (n=208)

Media source	Frequency	
	N	%
TV advertisements	55	26.44
Newspapers	41	19.71
Magazines	17	8.17
Google	31	14.90
Facebook	16	7.69
YouTube	39	18.75
Instagram	9	4.32

Most of the participants were worried about adverse drug effects and drug dependence and stayed away from pursuing self-medication (Table 5).

**Table 5:** Arguments against self-medication (n=212)

Reasons	Frequency	
	n	%
Concern about negative pharmacological (drug) effects	84	39.62
Concern for developing a drug addiction	36	16.98
Fear of a wrong diagnosis	15	7.07
Concern over the improper use of medications	23	10.84
Fear of dangerous medications	19	8.96
Inadequate medication knowledge	31	14.62
Others, if any	4	1.88

## Discussion

Self-medication is a widespread issue, and it is alarming when drug usage goes unreported. The prevalence and factors influencing self-medication were examined in this study, which was carried out in Saudi Arabia. Self-medication use was prevalent in the examined sample at 49.52%. In poor countries, consumers frequently self-medicate, and many prescription drugs may be purchased without a prescription [13-15]. The results of the current study will help the Saudi government develop measures for increasing public knowledge of the hazards associated with antibiotic consumption and determining the degree of antibiotic self-medication. In the present study, self-medication was shown to be prevalent in 49.52% of cases. This was significantly lower than studies conducted in Saudi Arabia by Almalki et al. [16] (67.7%), Alsaad et al. [17] (59%), Althagafi et al. [18] (76.9%), and Al-Ghamdi et al. [19] (81.3%), and higher when compared with Alghanim [20] (35.4%) and Albusalih et al. [21] (19.61%).

The frequency of self-medication practises varied greatly, ranging from 11.4% at the lowest end to 93.1% at the highest. Alzaharni et al. [22] reported the highest prevalence of self-medication practises, while Makeen et al. [23] observed the lowest prevalence. It is important to note that because the data were gathered after the COVID-19 pandemic, the prevalence that was found here may be higher than the prevalence of SM before the pandemic. This is probably because COVID-19 infection symptoms, including the flu, a cold, and fever, are typical and the most commonly reported self-treated symptoms in this study. Our study's findings showed that the majority of self-medicating people were men. The males in our study may have had more purchasing power and were employed, which are some of the possible causes. The non-Saudi population exhibits a higher tendency to self-medication in terms of nationality. This can be attributed to the high prevalence of self-medication among those, especially from Asian countries [24]. While one's own personal experience and a doctor's previous prescription were discovered to be the main sources for self-medication in fewer studies [25,26], advertisements and a variety of search engines were revealed to be the most common sources of information regarding self-medication in the current study.

Fever (49.51%) and the common cold (27.40%) were the most frequently reported causes of self-medication in the current study, which may be related to the fact that these two conditions affected more people than any other and that there is a widespread belief that they can be treated with over-the-counter medications without a doctor's consultation. Similar findings have been noted in earlier studies [27,28]. The majority of participants said that for minor illnesses, there was no need to visit the hospital for treatment. A pharmacy's proximity to their residences over a hospital is another factor. Cost-effectiveness is a strong argument as well, which makes sense given that buying drugs from a neighbourhood pharmacy is less expensive than getting advice from a doctor and paying a consultation charge. A crucial factor in this study is the kind of self-prescribed medication. Resistance to antibiotics is a very important issue. In line with the findings of a study conducted in India [29], paracetamol and cough syrups were the most commonly used classes of medications.

Compared to the Saudi population [16], where the frequency of antibiotic use without a prescription is 25.7%, just 7.21% of study participants in the current study used antibiotics without a prescription, which is in contrast with earlier studies in Egypt [30] (23.5%), Medina [26] (15.3%), Riyadh [31] (17.79%), and Qassim region [32] (13.8%) of Saudi Arabia. The Ministry of Health's awareness programmes on the use of antibiotics are responsible for the study's decreased rate. The majority of the participants practising self-medication reported that they have not faced any health issues due to it; 11% put an end to the medication after they experienced an issue. The biggest influences on people who self-medicate are TV ads (26.44%), newspapers (19.71%), and YouTube (18.75%). This result contrasts with that of the Ephrem et al. [10] study to determine how the media affects the usage of self-medication, in which individuals were more likely to use Instagram and Google to search for information on self-medication. The most common reason for not taking self-medication was the worry of adverse drug effects and drug dependence. Similar to our findings, a study done among a community in Addis Abeba, Ethiopia [33] discovered that fear of getting the wrong diagnosis, getting the wrong treatment, and experiencing adverse effects were common deterrents to SM practise.

Since the study relied on self-reported information on self-medication in the two months prior, recall bias is a possibility. Furthermore, although it was suggested that the subjects answer the questionnaire on their own, it was not completely possible to rule out that the participants would influence one another. If the study had included people from other countries as well, it might have been able to comprehend the socioeconomic, educational, and geographic influences and produce more generalizable conclusions. As certain ailments occur in cycles, a longer term may have been taken into account instead of two months.

## Conclusions

According to our study, Saudi residents self-medicate at a high rate. However, there were gaps in the population's understanding, attitude, and self-medication practises. Given that it may result in prescription overuse, the practise needs to be viewed as a public health issue. We urge greater public awareness of the value of adhering to label directions and the requirement of visiting a doctor before taking any medications. Additionally, it would be beneficial to use more precise language when discussing the amount and frequency of taking prescribed medications in order to increase understanding, particularly among patients with low education. The deceptive advertisements continue to circulate in the media, occasionally with endorsements from famous people. To safeguard innocent people from claims made by pharmaceutical firms with false claims or comments in newspapers, magazines, and electronic media, the government must impose severe regulations.
